# A commentary on ‘Core decompression combined with platelet-rich plasma-augmented bone grafting for femur head necrosis: a systematic review and meta-analysis’

**DOI:** 10.1097/JS9.0000000000001633

**Published:** 2024-05-17

**Authors:** Huihui Xu, Haipeng Huang, Chang Shao, Peijian Tong, Hongting Jin

**Affiliations:** a The First Affiliated Hospital of Zhejiang Chinese Medical University (Zhejiang Provincial Hospital of Chinese Medicine); b School of Basic Medical Sciences, Zhejiang Chinese Medical University, Hangzhou, Zhejiang, People’s Republic of China

*Dear Editor,*

We are interested in an article published in the *International Journal of Surgery* entitled ‘Core decompression combined with platelet-rich plasma-augmented bone grafting for femur head necrosis: a systematic review and meta-analysis’^1^. As we all know, as a progressive and destructive orthopedic disease, femur head necrosis mainly manifests as the collapse of the femoral head, which ultimately leads to hip pain and dysfunction and imposes a serious burden on the patients and society, so it is urgent to seek for an effective treatment method^2^. Surprisingly, the conclusions of this article suggested that core decompression and bone grafting combined with platelet-rich plasma (PRP) are superior to methods not combined with PRP in terms of function, pain relief, and imaging progression. In addition, it reduces the rate of treatment failure and the incidence of adverse events. This conclusive evidence indicated that we were one step closer to conquering femur head necrosis. However, while strongly commending the work, we have also identified several potential issues.

Firstly, regarding patient inclusion: of the 11 studies included, we found that the authors did not use the same criteria, with 7 studies using ARCO staging and 4 using Ficat staging, which, despite their similarities, are not identical^3^, so this may affect the accuracy of the results. In addition, we found that even among the studies that used the same staging, the stages were different. Specifically, of the 7 studies that used ARCO staging, 2 studies used stages I–II, 3 studies used stage II, and 2 studies used stages II–III. In contrast, of the 4 studies that used Ficat staging, 3 studies used stages I–II and 1 study used stages I–III. These would undoubtedly have a potential impact on the outcome indicators.

Secondly, regarding the preparation of PRP, the times of centrifugation varied among the included studies, in addition to the centrifugation time, centrifugation speed, and centrifugation radius. Nine studies used 2 centrifugations and 2 studies used a single centrifugation, so we can infer that the purity and composition of PRP obtained from each study were not the same. It has been demonstrated that 2 centrifugations are significantly better than a single centrifugation^4^. In addition, we found that the 2 studies that used single centrifugation used CaCl_2_ as an activator, whereas the remaining 9 studies did not use an activator, so we believe that it would have been more appropriate to exclude the 2 studies that used single centrifugation from this study.

Finally, regarding the choice of implanted bone, in the 11 studies included, the implanted bone was not the same, with three approaches being used: autologous bone, allograft bone, and artificial bone graft substitutes. In addition, the surgical methods used were also different, with 8 studies using the Phemister technique and 3 studies using the light bulb procedure, which undoubtedly also affected the accuracy of the results to some extent.

In summary, we would like to express our sincere gratitude to Zhu *et al*. for their meaningful work, which has been a major step forward in the fight against femur head necrosis. Our suggestions are only intended to further improve the already excellent research results, and we look forward to their future publications.

## Ethical approval

Our submitted manuscript does not involve any patients without the ethical approval document.

## Consent

Our submitted manuscript does not involve any patients without the written informed consent document.

## Sources of funding

Our submitted manuscript does not involve any sources of funding.

## Author contribution

H.X., H.H., and C.S.: participated in the original idea and writing the first draft; P.T. and H.J.: edited the final manuscript. All authors approved the last version of this manuscript.

## Conflicts of interest disclosure

There are no conflicts of interest.

## Research registration unique identifying number (UIN)

Not applicable.

## Guarantor

Peijian Tong, MD and Hongting Jin, MD.

## Data availability statement

The data in this manuscript can be obtained from the public internet.

## Provenance and peer review

Not applicable.

## References

[1] Zhu B Li J Li X . Core decompression combined with platelet-rich plasma-augmented bone grafting for femur head necrosis: a systematic review and meta-analysis. Int J Surg 2024;110:1687–1698. 38181110 10.1097/JS9.0000000000001028 PMC10942211

[2] Boontanapibul K Steere JT Amanatullah DF . Diagnosis of osteonecrosis of the femoral head: too little, too late, and independent of etiology. J Arthroplasty 2020;35:2342–2349. 32456965 10.1016/j.arth.2020.04.092

[3] Zhao D Zhang F Wang B . Guidelines for clinical diagnosis and treatment of osteonecrosis of the femoral head in adults (2019 version). J Orthop Translat 2020;21:100–110. 32309135 10.1016/j.jot.2019.12.004 PMC7152793

[4] Sheean AJ Anz AW Bradley JP . Platelet-rich plasma: fundamentals and clinical applications. Arthroscopy 2021;37:2732–2734. 34481615 10.1016/j.arthro.2021.07.003

